# Successful Regional Anesthetic for a Parturient with Moyamoya Syndrome

**DOI:** 10.1155/2020/1785041

**Published:** 2020-03-10

**Authors:** Anjalena Pasam, Akshatha Kamath, Hattiangadi Sangeetha Kamath, Joel Yarmush, Khaja Ahmed

**Affiliations:** Department of Anesthesiology, NYP-Brooklyn Methodist Hospital, Brooklyn, NY, USA

## Abstract

Anesthesia for Cesarean section could be challenging due to the physiological changes during pregnancy, but it can be more complicated if associated with sickle cell disease and moyamoya disease. The moyamoya syndrome is nothing but sickle cell disease complicated by cerebral vasculopathy. Incidence of moyamoya disease in the USA is 0.086/100,000 people. We report a case of a pregnant woman with sickle cell disease and moyamoya syndrome, who underwent a successful spinal epidural for primary cesarean section, with careful monitoring of blood pressure.

## 1. Introduction

Moyamoya disease has distinct clinical features due to the presence of longstanding narrowing of the internal carotid artery at its terminal part, with collateral circulation of anomalous blood vessels which appear on angiography as “moyamoya” in Japanese which means ‘puff of smoke [1, 2]. Patients who have sickle cell disease and neurovascular symptoms are found to have the moyamoya syndrome. We present a case of a parturient with a known history of sickle cell disease complicated with moyamoya syndrome (MMS) and scheduled for an elective cesarean section.

## 2. Case Report

**Figure 1:**
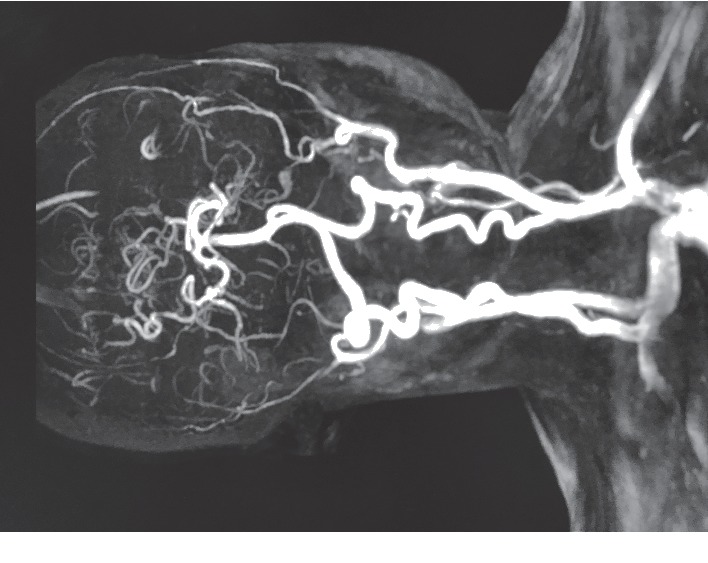
MRA showing bilateral internal carotid artery obliteration.

A 26-year-old primigravida, weighing 56 kilograms, native Puerto Rican woman with moyamoya syndrome, presented at 39 weeks of gestation for a scheduled primary cesarean section. She denied any obstetric-related complaints. Her history revealed frequent sickle cell crises requiring multiple exchange transfusions. She had a cerebrovascular accident six years ago with residual right limb weakness. She continued her daily aspirin dose of 81 mg throughout her pregnancy. On physical examination, the patient was an alert, oriented female with an unremarkable exam other than minimal right lower extremity weakness and a slight limp on gait. Ultrasonography revealed a single live fetus with a fetal heart rate of 130 bpm, in cephalic presentation, with fundal placenta. Laboratory studies were significant for elevated iron profile (serum iron level: 198 *μ*g/dL, TIBC: 252 *μ*g/dL, iron saturation: 78.5%, ferritin: 420.5 ng/mL, Hb-electrophoresis showing an Hb of 10 g/dl, and hematocrit: 31.4% with HbS of 57% and HbA of 31%). Past MRA revealed old infarcts in the left frontal lobe and complete occlusion of both ICAs (Figure 1). Surveillance four-vessel diagnostic cerebral angiography demonstrated that the sole supply of the anterior circulation was from the posterior circulation.

After consultation with the care team (obstetrician, neonatologist, internist, hematologist, neurosurgeon, and anesthesiologist), a controlled neuraxial anesthetic was chosen. Two large bore (18 gauge) IVs were started. A left radial arterial line was also started. Standard ASA monitors were applied, and the patient was prepared for the procedure in the sitting position. The patient was preloaded with 500 ml of normal saline, and a phenylephrine drip was primed and ready to administer. A combined spinal epidural anesthetic was performed. 1.6 ml of 0.75% bupivacaine with 25 mcg of fentanyl was administered intrathecally, and an epidural catheter was placed. It has been proven in prospective studies that low-dose spinal as a part of combined spinal epidural anesthesia is sufficient to provide adequate anesthesia for the procedure [3]. Phenylephrine drip was titrated to maintain the systolic blood pressure between 110 and 130 mmHg at all times. Procedure was uneventful. Blood loss was estimated to be 800 ml and was infused two liters of IV fluids; the patient was maintained normotensive, normocarbic, and normothermic, and neurological monitoring was performed throughout the procedure as the patient was awake as EEG was not available at that point in time. Additional 90 mg of epidural 3% chloroprocaine was supplemented as the patient reported pain after an hour throughout the procedure. A healthy baby with an APGAR score of 8 and 9 at 1 and 5 minutes, respectively, was delivered. Epidural morphine was administered after delivery of the baby for the postoperative pain control. Postoperatively, close monitoring of vitals along with frequent neurologic checks was performed with attention to hydration status and pain goals. Mother and baby were discharged home on the third postoperative day. She was provided with low molecular weight heparin for deep venous thrombosis prophylaxis.

## 3. Discussion

Due to characteristic progressive stenosis, cerebral flow is significantly decreased compared to normal healthy parturient. It is crucial to undergo evaluation of cerebral circulation prior to conception. If cerebral circulation is well maintained, the patient can safely undergo vaginal or caesarean delivery. In case of compromised cerebral circulation with symptoms, literature recommends prophylactic medical treatment [4]. For patients who do not respond to medical treatment, surgical options could be offered which include direct revascularization and indirect revascularization procedures. Elective cesarean section is the most chosen mode of delivery [5]; however, if vaginal delivery is opted, assisted delivery methods should be undertaken to avoid Valsalva during the bearing down.

Usually low-dose antiplatelet-like aspirin is prescribed for prophylactic prevention of strokes, which should not be a contraindication for neuraxial anesthesia [6]. Patients who report headaches benefitted from calcium channel blockers have the potential to cause hypotension [7]. Pregnancy-induced increase in estrogen and progesterone can accentuate vasodilation, and physiological increase in blood flow can lead to rupture of fragile blood vessels resulting in intracranial bleed, and this is more common in the antepartum period especially after 24 weeks of gestation [7]. Patient with sickle cell disease has a higher incidence of preterm labor, preeclampsia, and low birth weight babies.

Anesthetic management needs both careful evaluation and management of both sickle cell disease and moyamoya disease. Mismatch of cerebral blood flow, cerebral metabolic rate of oxygen (CMRO_2_), due to hypotension can aggravate ischemia, for example, dehydration secondary to vomiting in the last trimester of pregnancy and blood loss during delivery. Hypertension secondary to pain-induced increased sympathetic drive could lead to intracranial bleeding; hence, it is crucial to maintain hemodynamic stability and adequate pain control by early initiation of labor epidural with adequate hydration [8]. Ischemic episodes are more common after delivery especially the first week after delivery, due to hypovolemia which is secondary to blood loss, insensible loss, and hypercoagulable status. There have been studies/case reports which recommend increased oral intake of fluids after delivery to avoid hypovolemia [8]. Valsalva may play a sinister role during bearing down as it can increase the cerebral venous pressure and thereby decrease the cerebral perfusion pressure [9]. Hyperventilation can lead to hypocapnia causing cerebral vasoconstriction in turn decreasing cerebral perfusion pressure which could precipitate transient ischemic attacks/stroke [10]. Though neuraxial anesthesia is the preferred mode of anesthesia as per the current literature, the fear of accidental dural puncture resulting in the headaches should be done meticulously and if any dural puncture, be promptly treated with analgesics and fluid resuscitation. Neuraxial anesthesia provides an opportunity for neuromonitoring of the patient and hence may be preferred over the general anesthesia [11]. Moreover, neuraxial anesthesia itself can precipitate stroke due to intracranial hypoperfusion if severe hypotension.

## 4. Conclusion

It is essential to have a multidisciplinary team comprising of a hematologist, anesthesiologist, obstetrician, fetal medicine, and neurosurgeon as Moya Moya syndrome is rare. The bottom line is to optimize the patient's sickle cell disease, moyamoya disease and accordingly device a plan for intraoperative management to maintain an optimal end organ perfusion and oxygen delivery to the mother and fetus.
